# Narcotic Prescriptions following Knee and Shoulder Arthroscopy: A Survey of the Arthroscopy Association of Canada

**DOI:** 10.7759/cureus.7856

**Published:** 2020-04-27

**Authors:** Seper Ekhtiari, Nolan S Horner, Ajaykumar Shanmugaraj, Andrew Duong, Nicole Simunovic, Olufemi R Ayeni

**Affiliations:** 1 Orthopaedic Surgery, McMaster University, Hamilton, CAN; 2 Division of Orthopaedic Surgery, Department of Surgery, Mcmaster University, Hamilton, CAN; 3 Division of Orthopaedic Surgery, Department of Surgery, McMaster University, Hamilton, CAN

**Keywords:** narcotics, survey, prescription, patterns, arthroscopy, knee, shoulder, guidelines

## Abstract

Purpose

Canada has the second-highest opioid use in the world. Despite knee and shoulder arthroscopy being among the most commonly performed orthopaedic procedures, there exists little guidelines for pain management.

Methods

A survey was developed and distributed to members of the Arthroscopy Association of Canada. The objectives were: to understand opioid prescribing patterns after knee and shoulder arthroscopy, to determine if surgeons believe opioid over-prescription is an issue and to identify other pain management strategies surgeons are regularly using.

Results

A total of 38 responses were included (38.3%). Eighty-two percent of surgeons felt opioid over-prescription was an issue in arthroscopic surgery. The average post-operative knee or shoulder arthroscopy prescription included a total of 156 +/- 84.4 (0-400) mg of oral morphine equivalents (OMEs). Less than one-third of respondents (29%) had received formal peri-operative pain management training. Fifty-five percent of respondents felt that non-opioid medications do not provide adequate pain relief after arthroscopic surgery. Nearly all respondents (95%) stated they would change their prescription practice if high-quality evidence were to suggest that they should do so.

Conclusions

The majority of respondents identified opioid over-prescription as a problem after arthroscopic surgery. Surgeons are prescribing five times the amount of OMEs to patients that previous literature suggests the median patient uses after arthroscopic knee surgery. Surgeons generally state they would reduce or eliminate opioid prescriptions to arthroscopy patients if high-level evidence were to emerge suggesting that adequate pain control could be achieved without the use of narcotics.

## Introduction

Canada has the second highest per-capita opioid use in the world, with a threefold increase in the rate of prescription opioid use over the past ten years [1-2]. The Government of Canada has declared that “Canada is facing an opioid crisis…the growing number of overdoses and deaths caused by opioids…is a public health emergency” [3]. The problem appears to be worsening: In 2018, there were 4,588 opioid-related deaths across Canada, representing a 152% increase compared to the 3,023 deaths in 2016 [4]. Opioids, though effective for short-term pain relief and severe pain near the end of life, are high-risk medications for addiction, tolerance, withdrawal, and fatal overdose [5].

Orthopaedic surgeons prescribe more opioid medications, including long-acting narcotics, than any other surgical specialty [6-7]. A recent database study across various surgical specialties found that 94% of patients undergoing elective surgery received opioid prescriptions at discharge, and that all orthopaedic surgery patients were well in excess of the recommended opioid prescription guidelines and had relatively high refill rates [8]. Furthermore, the vast majority (67-92%) of patients receiving prescription opioids after surgery report having unused narcotics, and only a quarter of these patients store their excess medications in locked containers [9]. In other areas of the world, patients take far fewer opioids compared with American and Canadian patients, but report similar pain and satisfaction with pain management [10].

In 2006 knee and shoulder arthroscopy were found to be among the most commonly performed orthopaedic surgery procedures [11]. Despite this, there are no clinical practice guidelines for pain management following these common procedures. Locally, it is routine practice to prescribe narcotic medications after arthroscopic surgery, and there is no standardized process for assessing post-operative opioid use. Thus, this survey was conducted with the following objectives: 1) To establish an understanding of narcotic prescription patterns by surgeons for post-operative pain control for patients undergoing elective knee and shoulder arthroscopy across Canada, 2) to determine if Canadian surgeons believe they or other surgeons are on average over-prescribing narcotic medications after knee and shoulder arthroscopy, and 3) to identify what non-opioid pain management strategies surgeons are regularly using after knee and shoulder arthroscopy.

## Materials and methods

Questionnaire development

We assembled a focus group consisting of an author with statistical analysis experience and orthopaedic surgeons who perform knee and shoulder arthroscopy regularly. An initial draft of the questionnaire was made by one of the authors (SE) and then distributed sequentially to the focus group consisting of two orthopaedic surgery residents, a statistician, and a fellowship trained arthroscopic surgeon for feedback on the content, clarity and comprehensiveness of the survey. Questions were developed to examine respondents’ demographic characteristics, current opioid prescribing patterns after knee and shoulder arthroscopy, non-opioid pain management strategies surgeons are currently utilizing after knee and shoulder arthroscopy and surgeons’ perceptions of the opioid crisis in Canada. The final questionnaire, which had approval from the members of the initial focus group, consisted of 23 questions using multiple choice, Likert scales, limited commentary, and open responses. A complete version of the survey can be found in Appendix 1. This study received ethics approval from the Hamilton Integrated Research Ethics Board (REB No. 7560) on July 18, 2019.

Questionnaire administration

The survey was distributed to all physician members of the Arthroscopy Association of Canada (AAC) electronically through SurveyMonkey (N = 99). The survey was initially distributed on July 23, 2019, and reminder e-mails were sent on August 14, 2019 and September 6, 2019. Responses for the survey were closed on September 13, 2019. Responses were restricted to a single response per individual. Individuals who stated that they did not perform arthroscopic knee or shoulder surgery were excluded from the study. After the end date of the survey, the data from SurveyMonkey was exported to a secure Excel spreadsheet (Version 14.7, Microsoft, Redmond, Washington, USA) prior to data analysis.

Statistical analysis

The statistical analysis plan was established a priori and contained within the REB submission. All responses were reported in descriptive statistics, using proportions or mean ± standard deviation (SD) as appropriate. Medication doses were converted into oral morphine equivalents (OMEs) using the Oregon Pain Guidance online calculator tool [12]. Some Likert scale categories were collapsed as appropriate if they shared directionality (e.g. combining responses of “Strongly Agree” and “Agree” together).

## Results

A total of 39 surveys were completed (39.4% response rate). Of these surveys, one participant indicated that they do not perform knee or shoulder arthroscopy and therefore was excluded. Thus, 38 responses were included in the final analysis. Responses to each of the survey questions can be found in Appendix 2.

Characteristics of the respondents

All respondents were physician members of the AAC and were fully licensed orthopaedic surgeons in independent practice. All ten Canadian provinces were represented, with Alberta (29%) and Ontario (24%) being most common (Table 1). Primary practice location was divided between academic (53%) and community centres (47%). The most common caseloads for knee arthroscopy were 51-100 cases per year and > 200 cases per year (32% each), and for shoulder < 50 cases per year (29%).

**Table 1:** Primary practice locations of respondents ^a^ Denominator = 38

Province	N	%^a^
Alberta	11	29.0%
British Columbia	6	15.8%
Manitoba	2	5.3%
New Brunswick	2	5.3%
Newfoundland and Labrador	1	2.6%
Nova Scotia	2	5.3%
Ontario	9	23.7%
Prince Edward Island	1	2.6%
Quebec	2	5.3%
Saskatchewan	2	5.3%

Peri-operative pain management strategies

Most respondents routinely utilized cryotherapy after knee (79%) and shoulder (68%) arthroscopy. Over half of all respondents also performed an intra-articular injection at the conclusion of their knee arthroscopy cases (55%), compared to 40% who did so for the shoulder. Routine nerve blocks were more common in shoulder arthroscopy (42%) compared to knee arthroscopy (29%).

Practice patterns

The respondents reported that on average, 88% (standard deviation = 23%) of their post-arthroscopy patients received a prescription for an opioid medication. Tylenol #3 was the most commonly prescribed first-line opioid (31%), followed by hydromorphone (25%) and tramadol (31%) (Table 2). Over one-third of all respondents also prescribed a second opioid medication (34%). The average prescription was for a total of 156 +/- 84.4 mg of OMEs (i.e. roughly equivalent to a prescription for 31 tabs of 5 mg morphine). Surgeons’ routine prescription after knee or shoulder arthroscopy ranged from 0 to 400 mg of OMEs. Only one respondent stated that they did not regularly prescribe opioids after knee or shoulder arthroscopy.

**Table 2:** First line opioid medications prescribed ^a^ Denominator = 36

Medication	N	%^ a^
Morphine	1	2.8%
Oxycodone	6	16.7%
Percocet	1	2.8%
Hydromorphone	9	25.0%
Tylenol #3	11	30.6%
Tramadol	8	22.2%

**Figure 1:**
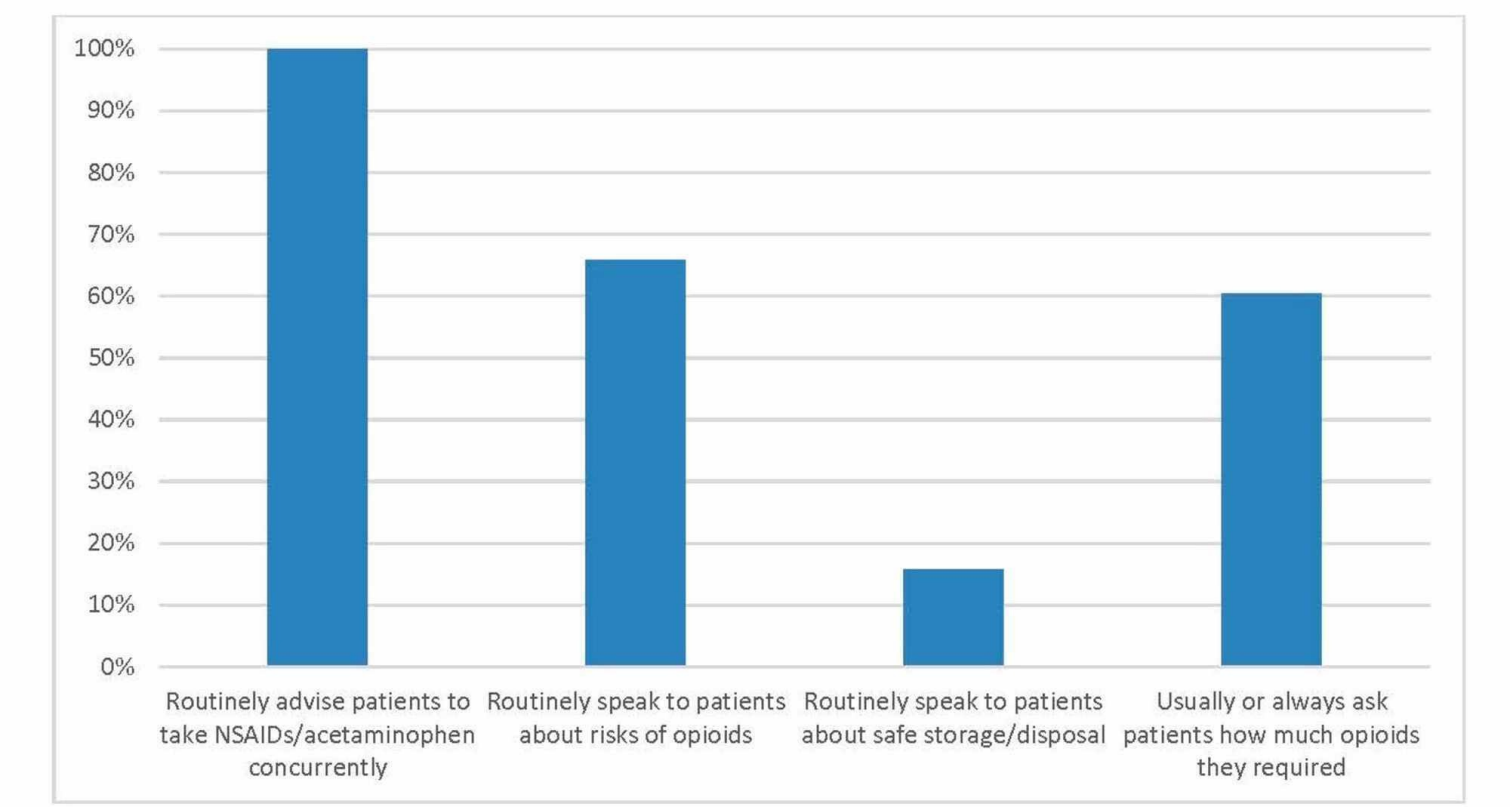
Practice patterns regarding counselling patients on opioid use

All respondents stated that, in the absence of any contraindications, they routinely advise their patients to take non-steroidal anti-inflammatories (NSAIDs) and acetaminophen concurrently with any opioid medication (Figure 1). Two-thirds of respondents (66%) stated that they routinely speak to their patients about the risks of opioids, while only 16% discussed safe storage and disposal practices. Over half (61%) of respondents “usually” or “always” ask their patients at follow-up visits about how much opioids they required. Respondents estimated that 12.1 ± 11% of their patients required at least one refill prescription.

Peri-operative pain management training

Less than one-third of all respondents (29%) had received formal peri-operative pain management training. Among those who had received such training, 91% said it had “some” or “a great deal” of influence on their prescription patterns. As well, 79% of those who had not received any formal training felt that such training would be useful to them.

Attitudes regarding opioid prescription in orthopaedics

**Figure 2:**
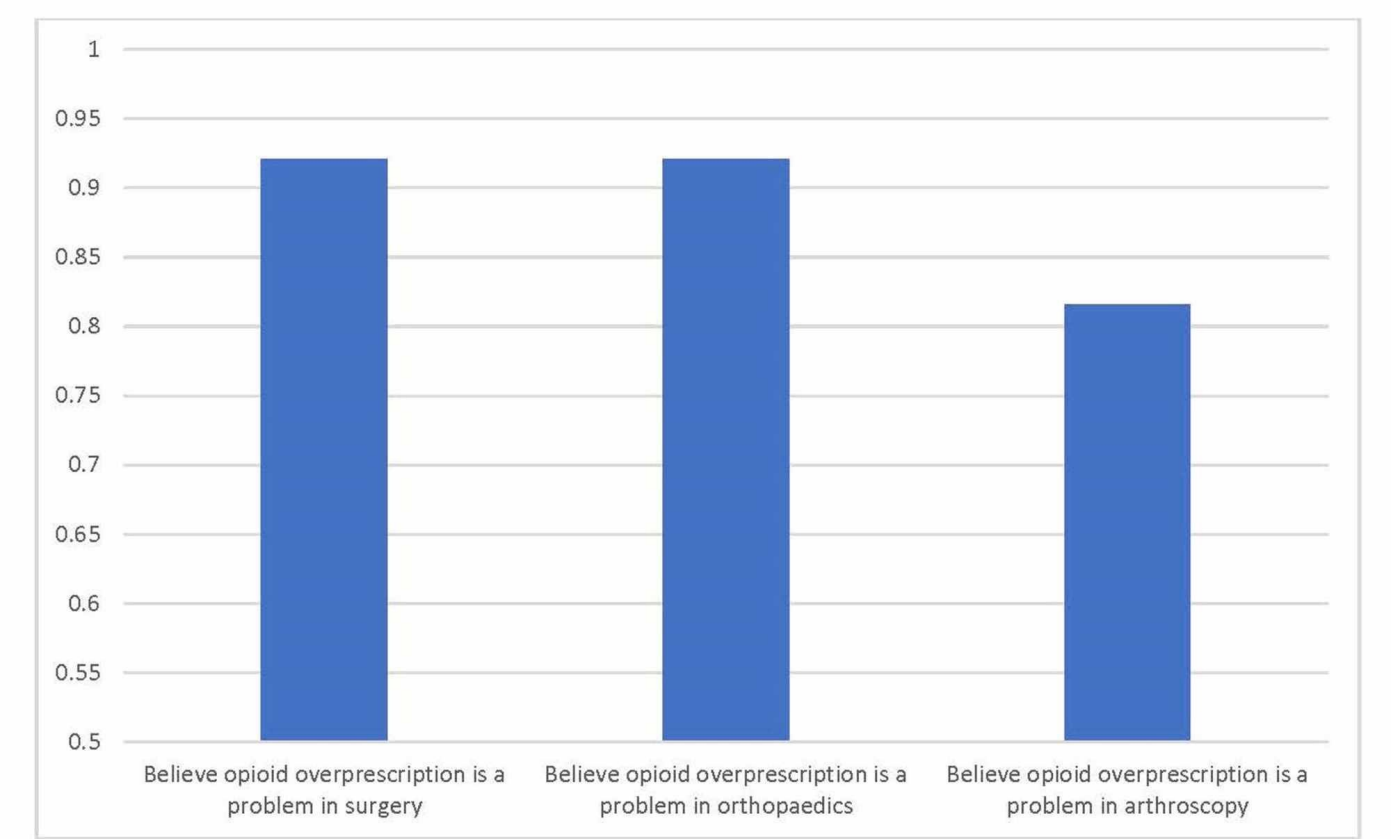
Attitudes towards opioid over-prescription as a problem

Nearly all (92%) respondents agreed that opioid over-prescription is a problem in surgery generally, and orthopaedics specifically (Figure 2). A slightly lower proportion (82%) believed that it was a problem in arthroscopy as well. Nearly two-thirds of surgeons (63%) felt that they personally over-prescribed opioids for their arthroscopy patients, with uncertainty about the efficacy of other analgesic modalities being the primary reason (38%). Forty-five percent of respondents felt that non-opioid medications would provide adequate pain relief after arthroscopic surgery, while 55% disagreed. Similarly, 42% of surgeons thought that patients would be accepting of receiving only non-opioid medications after discharge from arthroscopy, while 37% thought that patients would be resistant to this idea. Finally, nearly all respondents (95%) stated that they would change their current practice if high-quality evidence were to suggest that they should do so.

## Discussion

The key finding in our survey was that 88.1% of surgeons are currently prescribing opioid pain medications after elective knee and shoulder arthroscopy. Furthermore, 92% of surgeons believe that opioid over-prescription in orthopaedic surgery is a significant issue and over 80% said that they thought it was an issue in arthroscopy specifically. However, there is uncertainty amongst surgeons as to whether non-opioid pain medications such as acetaminophen and NSAIDs alone would provide adequate pain relief to patients after these surgeries. Furthermore, the effects of NSAIDs on bone healing rates can possibly influence the post-operative pain management prescribed by surgeons [13]. To the best of the authors’ knowledge, there remains a dearth of knowledge in the utility of non-opioid pain medications for post-operative pain control after elective knee or shoulder arthroscopy. In particular, studies focussing on pain management strategies for partial meniscectomies and chondroplasties exist, with no studies focussing on other arthroscopic surgeries for the knee or shoulder. One prospective cohort study investigated the use of non-opioid pain management (i.e. NSAIDs or acetaminophen) and found that 82% of patients undergoing arthroscopic partial meniscectomy and/or chondroplasty have satisfactory pain control [14]. Furthermore, a case series of 175 patients found that preoperative resilience scores does not correlate with a non-opioid pain regimen following partial meniscectomy and/or chondroplasty [15].

A recent prospective study found that knee arthroscopy patients only used a median of seven 5 mg hydrocodone pills (i.e. total 35mg of OMEs) and 88% percent of patients had a surplus of unused opioids [16]. Our study found that Canadian surgeons were on average prescribing 156 +/- 84.4 mg of OMEs to patients, i.e. nearly five times as much as the median patient requires. Respondents stated that they prescribed as much as 400 mg of OMEs, i.e. over five to ten times the median patient requirement. This suggests that surgeons are likely prescribing significantly more opioids than patients require, at least for elective knee arthroscopy. In fact, 63% of respondents to our survey stated they felt they were knowingly over-prescribing opioids post-operatively. Furthermore, surgeons were rarely (16%) addressing safe storage and disposal of excess opioids to patients. The authors of the current study recommend safe storage by storing the medications away from children, ideally locked [17]. Meanwhile, disposal of excess opioids at a community pharmacy for proper disposal methods is recommended [17].

Post-operative opioid prescriptions represent only one of several ways to manage patients’ post-operative pain. There are many studies supporting the use of pain control adjuncts such as intra-articular injections, nerve blocks, and cryotherapy after knee and shoulder arthroscopy [18-20]. Our survey found that cryotherapy was the most common adjunct being used to help reduce post-operative pain after knee and shoulder arthroscopy. Intra-articular injections were being used by approximately half of the surgeons after these procedures, and nerve blocks were the least frequently used adjunct. Improved uptake of these pain control adjuncts would likely result in a decrease in the amount of post-operative opioids that patients require for pain control. More work is necessary to determine why many surgeons chose not to adopt these other pain control modalities. It can be hypothesized that this may be a result of surgeon preference, though evidence may suggest the effectiveness of other pain control modalities.

Approximately 60% of surgeons responded to the survey stating that they routinely discussed the risks of opioid use with their patients prior to prescribing them. Hartford et al. recently published their study where patients were prospectively enrolled after either open hernia repair or laparoscopic cholecystectomy and were either treated with standard pain management strategies or with a multimodal intra- and postoperative analgesic bundle, including promoting co-analgesia, opioid-reduced prescriptions, and patient education [21]. They found that neither group reported significantly different postoperative pain scores, but patients treated with the multimodal analgesic bundle were on average prescribed half the number of OMEs with no significant difference in prescription renewal rates. Syed et al. also found that pre-operative opioid education for patients undergoing arthroscopic rotator cuff repair significantly reduced the number of OMEs patients consumed at a three-month follow-up [22]. These studies suggest that multimodal approaches that include a focus on patient and provider education on pain management strategies may prove to be useful to orthopaedic surgeons attempting to reduce their rates of opioid prescribing.

The main strength of this study was the fact that the survey received responses from surgeons of a variety of practice types including academic and community surgeons across Canada. Furthermore, the survey was designed and piloted by a test group of orthopaedic surgeons. Limitations of this survey include the possible variability amongst the amount of post-operative pain different arthroscopic knee and shoulder procedures cause. For instance, one study showed that patients treated with a meniscal repair required significantly more opioids post-operatively than the average knee arthroscopy patient [16]. Furthermore, despite our moderate response rate, the absolute number of survey responses was low due to the relatively low number of surgeons in Canada compared to other countries such as the United States of America. As such, this survey is at risk of sampling bias due to the moderate response rate.

Most Canadian surgeons state that opioid over-prescription in arthroscopic surgery is an issue, however, there is clinical equipoise amongst respondents as to whether non-opioid prescriptions would provide adequate pain relief to patients after elective knee and shoulder surgery. Furthermore, surgeons indicated that they are willing to change their opioid-prescribing practice if strong evidence suggests emerges to suggest that they are not necessary. Therefore, high-quality studies are needed to determine how much, if any, opioids patients actually require to manage post-operative pain after elective knee and shoulder arthroscopy. One respondent in our survey remarked that official AAC guidelines on opioid prescribing would be useful. Future high-quality studies should be used to form evidence-based guidelines surrounding opioid prescriptions after elective knee and shoulder arthroscopy.

## Conclusions

The majority of respondents identified opioid over prescription as a problem after arthroscopic surgery. Surgeons are prescribing five times the amount of OMEs to patients that previous literature suggests the median patient uses after arthroscopic knee surgery. Surgeons generally state they would reduce or eliminate opioid prescriptions to arthroscopy patients if high level evidence were to emerge suggesting that adequate pain control could be achieved without the use of narcotics.

## Appendices

Appendix 1

Opioid Prescriptions following Knee and Shoulder Arthroscopy: A Surgeon Survey

Introduction

Dear Doctor,

The purpose of this research project is to learn about the practice patterns and perspectives of Canadian arthroscopists regarding the use of opioids post-operatively.

This is a research project being conducted by Dr. Femi Ayeni, orthopaedic surgeon, at McMaster University. You are invited to participate in this research project because you are identified as a member of the Arthroscopy Association of Canada.

Your participation in this research study is voluntary. You may choose not to participate. If you decide to participate in this research survey, you may withdraw at any time. If you decide not to participate in this study or if you withdraw from participating at any time, you will not be penalized.

The procedure involves filling an online survey that will take approximately seven minutes. Your responses will be confidential and we do not collect identifying information such as your name or email address. The survey questions will be about your demographics, current practice patterns, and beliefs and attitudes regarding opioid use after knee and shoulder arthroscopy.

We will keep your information confidential. All data is stored in a password protected electronic format. To help protect your confidentiality, the surveys will not contain information that will personally identify you. The results of this study will be used for scholarly purposes only and may be shared with study investigators.

We realize that knee and shoulder arthroscopy are different, and that each procedure is different (e.g. reconstructive vs. not). We want to get a global sense of all-comers in arthroscopic surgery, thus please answer questions with this frame of mind.

If you have any questions about the research study, please contact Dr. Femi Ayeni (contact information in cover letter). This research has been reviewed and approved according to the Hamilton Integrated Research Ethics Board procedures for research involving human subjects.

By clicking 'Next', you agree to participate in this study according to the above terms.

Opioid Prescriptions following Knee and Shoulder Arthroscopy: A Surgeon Survey

Screening

* 1. Are you a fully trained orthopaedic staff surgeon currently in independent practice (i.e. not fellowship or residency)?

Yes

No

* 2. Do you regularly perform knee and/or shoulder arthroscopy?

Yes

No

Opioid Prescriptions following Knee and Shoulder Arthroscopy: A Surgeon Survey

Demographics

* 3. In which province or territory do you practice?

Alberta

British Columbia

Manitoba

New Brunswick

Newfoundland and Labrador

Northwest Territories

Nova Scotia

Nunavut

Ontario

Prince Edward Island

Quebec

Saskatchewan

Yukon

* 4. Do you mainly operate at an academic centre or a community centre?

Academic centre

Community centre

* 5. What is your average annual knee arthroscopy case volume?

Number of Knee Cases

<50 cases per year

51-100 cases per year 101-200 cases per year

>200 cases per year N/A, do not perform

* 6. What is your average annual shoulder arthroscopy case volume?

Number of Shoulder Cases

<50 cases per year

51-100 cases per year 101-200 cases per year

>200 cases per year N/A, do not perform

Opioid Prescriptions following Knee and Shoulder Arthroscopy: A Surgeon Survey

Practice Patterns

7. Which of the following peri-operative pain management techniques do you routinely utilize (check all that apply)?

Knee Arthroscopy                                                                 Shoulder Arthroscopy

Cryotherapy (simple ice packs or cold therapy units such as Game Ready®)

Intra-articular pain pump

Post-arthroscopy intra- articular injection

Nerve block

Other (please specify)

* 8. What percentage of your patients undergoing knee or shoulder arthroscopy receive a prescription for an opioid pain medication upon discharge?

0-100%

9. Please specify your TYPICAL (i.e. most common) post-operative opioid prescription for patients undergoing knee or shoulder arthroscopy. PLEASE fill out QUANTITY and REFILLS.

NOTE: please ONLY use multiple lines if you ROUTINELY prescribe more than one medication, otherwise simply list your most common.

MEDICATION       DOSE           UNITS                           ROUTE                           FREQUENCY                  REGULAR OR PRN

Medication 1

Medication 2

Medication 3

Medication 4

Medication 5

Other (please specify)

10. Do you routinely advise your patients undergoing knee or shoulder arthroscopy that they may take acetaminophen and/or NSAIDs concurrently and in addition to opioids to manage their post-operative pain (in the absence of any contraindications)?

Yes

No

11. If you prescribe opioids after knee and shoulder arthroscopy, do you routinely speak to your patients about the risks of these medications?

Yes

No

N/A, I do not prescribe opioids after knee and shoulder arthroscopy

12. If you prescribe opioids after knee and shoulder arthroscopy, do you routinely speak to your patients about safe storage and disposal of their opioid medications?

Yes

No

N/A, I do not prescribe opioids after knee and shoulder arthroscopy

13. At post-operative clinic visits, how often do you ask patients about the quantity of opioids they required?

Always 

Usually

Sometimes

Rarely

Never

N/A, I do not prescribe opioids after knee and shoulder arthroscopy

Opioid Prescriptions following Knee and Shoulder Arthroscopy: A Surgeon Survey

Training, Beliefs, and Attitudes

14. How often do you estimate that your patients who are post-op from knee and shoulder arthroscopy ask for additional opioids beyond the initial prescription you provide?

0                                                            %                                                         100

15. Throughout your training or since starting your practice, have you ever received formal training in peri-operative pain management?

Yes

No

16. If yes, how much influence does this training have in your current prescription patterns?

A great deal of influence

Some influence

N/A

A little influence

Comments

17. If no, how useful do you think such training would be to your practice?

Very useful

Not useful at all

Somewhat useful

N/A

Slightly useful

Comments

18. Do you believe opioid over-prescription is an issue in:

Yes

No

Surgery (all specialties)?

Orthopaedics (all subspecialties)?

Arthroscopy?

19. Do you believe that non-opioid medications would provide adequate post-op analgesia for the majority of patients having shoulder or knee arthroscopy?

Yes

No

20. Do you believe that, on average, you prescribe more than a sufficient number of opioids for your post- op arthroscopy patients?

Yes 

No 

N/A

21. If yes, what is your primary reason for prescribing more opioids than you believe to be necessary?

Habit

Patient expectation/demand

Uncertainty about the efficacy of non-opioid pain medications (NSAIDs, Acetaminophen, etc.)

Consistency with the practice of other colleagues

Other (please specify)

22. How accepting do you think your average knee or shoulder arthroscopy patient would be of receiving only non-opioid medications post-operatively?

Very accepting

Somewhat accepting

Neutral

Other (please specify)

Somewhat resistant

Very resistant

* 23. If high-quality evidence reveals that patients would be satisfied with very limited or no opioids after knee or shoulder arthroscopy, would you change your practice accordingly?

Yes

No

N/A, I do not prescribe opioids after knee and shoulder arthroscopy Please elaborate as needed

24. If you have any other thoughts or comments on this topic, please provide them here:

Appendix 2

**Table 3:** Detailed survey results

	N	%
Type of Practice:		
Academic Centre	20	52.6%
Community	18	47.4%
Average Annual Case Volume (Knee):		
<50 cases per year	3	7.9%
50-100 cases per year	12	31.6%
101-200 cases per year	8	21.1%
> 200 cases per year	12	31.6%
Do not perform	3	7.9%
Average Annual Case Volume (Shoulder):		
<50 cases per year	11	28.9%
50-100 cases per year	7	18.4%
101-200 cases per year	8	21.1%
> 200 cases per year	7	18.4%
Do not perform	5	13.2%
Routinely use the following (Knee):		
Cryotherapy	30	78.9%
Intra-articular pain pump	0	0%
Post-arthroscopy intra-articular injection	21	55.3%
Nerve block	11	28.9%
Routinely use the following (Shoulder):		
Cryotherapy	26	68.4%
Intra-articular pain pump	0	0%
Post-arthroscopy intra-articular injection	15	39.5%
Nerve block	16	42.1%
At post-operative visits, how often do you ask patients about how much opioids they required?		
Always	6	15.8%
Usually	17	44.7%
Sometimes	8	21.1%
Rarely	5	13.2%
Never	1	2.6%
N/A, I do not prescribe opioids	1	2.6%
Among those how had received peri-operative pain management training, how much influence does this training have on your practice?		
A great deal of influence	5	41.7%
Some influence	6	50.0%
A little influence	1	8.3%
None at all	0	0%
How useful do you think peri-operative pain management training would be to your practice?		
Very useful	14	42.4%
Somewhat useful	12	36.4%
Slightly useful	7	21.2%
Not useful at all	0	0%
For those who believe they over-prescribe opioids after arthroscopy, what is the reason for this?		
Habit	2	8.3%
Patient expectations	7	29.2%
Uncertainty about efficacy of non-opioids	9	37.5%
Consistency with the practice of colleagues	1	4.2%
Other	5	20.8%
How accepting do you think patients would be of non-opioid protocols after arthroscopy?		
Very accepting	2	5.3%
Somewhat accepting	14	36.8%
Neutral	5	13.2%
Somewhat resistant	11	29.0%
Very resistant	3	7.9%
Other	3	7.9%
